# Supplementary material for: Exceedingly High Performance Top-Gate P-Type SnO Thin Film Transistor with a Nanometer Scale Channel Layer
Source: Nanomaterials (Basel). 2021 Jan 3;11(1):92. doi: 10.3390/nano11010092 (PMC7823917; doi:10.3390/nano11010092)
Supplement: Supplementary file 1 [file nanomaterials-11-00092-s001.pdf]

nanomaterials

Article

Exceedingly High Performance Top-Gate P-Type SnO Thin Film Transistor with a Nanometer Scale Channel Layer

Te Jui Yen [1], Albert Chin [1,*] and Vladimir Gritsenko [2,3,4]

[1] Department of Electronics Engineering, National Chiao Tung University, Hsinchu 300, Taiwan
[2] Rzhanov Institute of Semiconductor Physics, Siberian Branch, Russian Academy of Sciences, Novosibirsk, Russia
[3] Novosibirsk State University, Novosobirsk, Russia
[4] Novosibirsk State Technical University, Novosibirsk, Russia
* Correspondence: email: achin@nctu.edu.tw, Tel.: +886-3-5731841

Received: date; Accepted: date; Published: date

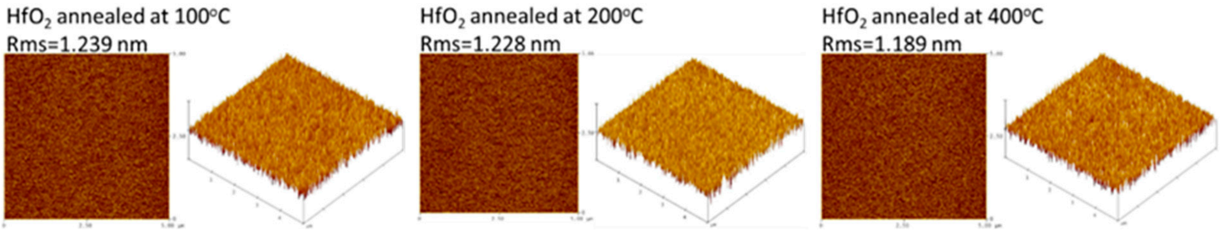

(a) (b) (c)

Figure S1. The surface roughness analysis of HfO_2 annealed at (a) 100 °C, (b) 200 °C and (c) 400 °C through AFM.

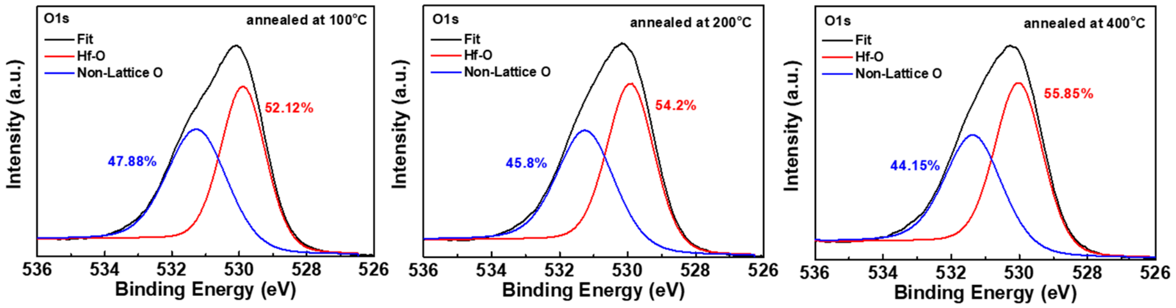

(a) (b) (c)

Figure S2. The O_{1s} spectra of HfO_2 films annealed at (a) 100°C, (b) 200°C and (c) 400°C.
